# CYP2A6 Polymorphisms May Strengthen Individualized Treatment for Nicotine Dependence

**DOI:** 10.1155/2015/491514

**Published:** 2015-04-28

**Authors:** Yawo Mawuli Akrodou

**Affiliations:** Department of Public Health, College of Health Sciences, Walden University, Minneapolis, MN 55401, USA

## Abstract

Each *CYP2A6* gene variant metabolizes nicotine differently depending on its enzymatic activities. The normal nicotine metabolizer CYP2A6^*^1A is associated with high scores of nicotine dependence (5–10) on the Fagerström Test for Nicotine Dependence (FTND) scale because it encodes for enzymes that catalyze nicotine 100%. Slow nicotine metabolizers (i.e., CYP2A6^*^1H, CYP2A6^*^4A, CYP2A6^*^9, and CYP2A6^*^12A) are associated with underrated nicotine metabolizing activity (50%–75%), linking them to low scores for nicotine dependence (0–4) on the FTND scale. In a clinical trial involving the use of bupropion, people who were carriers of slow nicotine metabolizers were found to have a tendency to maintain abstinence 1.7 times longer than people with normal nicotine metabolizers. An overview of CYP2A6 polymorphism enzymatic activities in nicotine dependence etiology and treatment revealed that slow nicotine metabolizers may strengthen the individualized treatment of nicotine dependence.

## 1. Introduction

*CYP2A6* polymorphism consists of alleles with variable structures, ranging from deleted alleles (*CYP2A6* ^*∗*^ *2A/CYP2* ^*∗*^ *4A*) to fully functioning alleles (*CYP2A6* ^*∗*^ *1A*), which determine the role that their level of enzymatic activity plays in nicotine etiology and the impact they have on nicotine dependence treatment [1, 2]. The nicotine candidate gene* CYP2A6∗1A* has normally high enzymatic activity in nicotine digestion and is responsible, in part, for the high prevalence of smoking by predisposing people to nicotine addiction [3, 4], causing more than 5 million [5, 6] smoking-related deaths and disabilities each year. On the other hand, slow nicotine metabolizers may realize potential clinical benefits since they metabolize less nicotine [1]. This paper reviews the ability of* CYP2A6* polymorphisms to digest nicotine differentially and investigates their impact on nicotine dependency treatment in the context of personalized treatments for nicotine dependence.

## 2. Study Background and Theoretical Foundation

The enzymatic activities of the normal nicotine metabolizer* CYP2A6* ^*∗*^ *1A* can be considered to be responsible, in part, for the high prevalence of nicotine dependence due to* CYP2A6* ^*∗*^ *1A*'s excessive metabolism of inhaled nicotine [1]. The slow nicotine metabolizer alleles of* CYP2A6* catalyze less nicotine and positively influence nicotine-dependence treatment [7]. Nevertheless, current pharmacotherapy treatments are not tailored to the genetic makeup of nicotine-dependent people, limiting the odds of successful treatment outcomes in nicotine dependence. Unfortunately, more than 5 million deaths annually are linked to tobacco-related diseases. In the United States (US) alone, the smoking prevalence is still 20%; moreover, approximately 400,000 deaths each year are associated with tobacco-related diseases [5, 6], thereby compelling a search for a novel treatment for nicotine dependence.

In general, the implication of genes in addiction etiology and their possible use in treatment should be considered to individualize addiction treatment. Behavioral Genetic Theory (BGT) underlines the existence of a correlation between genes and addresses the role that exposure to environmental agents [8–12] plays in an individual's predisposition to develop an addiction or in preventing an individual from becoming addicted to a substance. For example, the interaction between nicotine and the* CYP2A6* polymorphism* CYP2A6* ^*∗*^ *1A* (normal nicotine metabolizer) leads to nicotine addiction [13].

**Figure 1:**
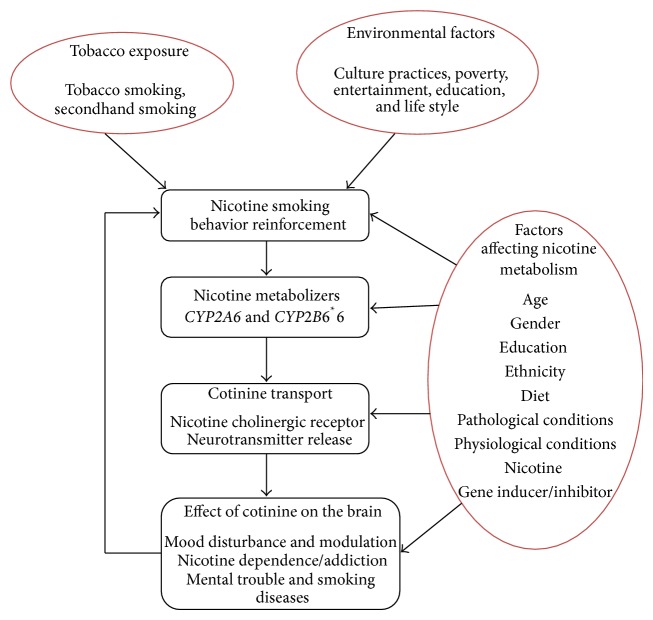
Factors influencing nicotine behavior and metabolism rate.* Note.* Environmental, cultural, and social factors may influence the development of nicotine intake, but nicotine metabolism is best modulated by* CYP2A6* genes which release cotinine into blood to reach brain inducing pleasurable feeling for smokers.

The capability of genes to metabolize drugs can strengthen or weaken nicotine dependence treatment by influencing nicotine pharmacodynamics and pharmacokinetics [5, 14]. Nicotine is an environmental agent; when it interacts with specific candidate nicotine genes, it can play an important role in the development of nicotine dependence [14], as shown in Figure 1. The normal nicotine metabolizer* CYP2A6* ^*∗*^ *1A* is one of the first biological mediators of nicotine digestion [15]. The suppression or alteration of the* CYP2A6* ^*∗*^ *1A* gene variant, which excessively metabolizes nicotine, should lead to the excretion of undigested nicotine in the urine by blocking cotinine from reaching the brain, thereby slowing down nicotine dependence [15, 16]. In fact, a slow nicotine metabolizer, such as* CYP2A6* ^*∗*^ *4A*, has a deleted allele of* CYP2A6*, which does not metabolize nicotine. It disrupts the normal digestion process of nicotine, which is simply evacuated instead. Consequently, slow metabolizers have potential health benefits in nicotine dependence treatment for carriers [13, 16–20], acting as a type of nicotine-dependence treatment drug because the pharmacologic nicotine dependence treatment strategy consists of injecting small doses of nicotine into the blood, which limits the volume of nicotine needed to excite the brain [7, 21, 22].

## 3. Limitations of Current Nicotine-Dependence Therapies

Smoking cessation has major health benefits for men and women of all ages. However, a long-term cure for nicotine dependence remains difficult to achieve since more than 80% of moderate-to-heavy smokers who seek treatment relapse within 1 year [23]. Although many methods have been developed to aid smoking cessation (e.g., pharmacotherapy and behavioral counseling), currently available nicotine dependence treatments are still rather limited in efficacy [23]. While smoking behavior is sustained biologically by candidate nicotine genes, such as the normal nicotine metabolizer* CYP2A6* as well as other candidate nicotine genes, including dopamine receptors and CYP2B6 gene variants [24, 25], information about these genes is not used in current nicotine dependence treatment.

There is a specific mass (1.0–1.5 mg) of nicotine that should be metabolized continuously while smoking in order to allow the tobacco user to enjoy a pleasurable feeling (triggered by the impact that nicotine has on the brain), which leads to his/her addiction [26, 27]. According to Hukkanen et al. [22], 1.0–1.5 mg of nicotine is readily absorbed while smoking and easily passes through the lung alveoli membrane because nicotine is a weak base with a pKa = 0.8. The absorption of nicotine through the lung membrane is facilitated by the pH level, and then nicotine is transported by the bloodstream to the brain within 10 seconds, where it induces pleasurable sensations. The mechanism of nicotine digestion occurs with the mediation of multiple candidate nicotine genes, but the most relevant nicotine gene metabolizers (and the most well-studied) are the polymorphic alleles of the* CYP2A6* gene.

The role of* CYP2A6* polymorphism is a determinant in the development of nicotine dependence and its treatment [4]. In treatment, a relapse of the nicotine addiction mechanism occurs because nicotine abstinence reduces dopamine release, triggering nicotine withdrawal symptoms, such as unpleasant mood states (irritability, anxiety, depression, and restlessness), poor concentration, hunger, sleep disturbance, and a craving for nicotine [28]. These withdrawal symptoms often defy the efficacy of current pharmacotherapies that use bupropion, nortriptyline, clonidine and nicotine patches, nicotine gum, inhalers, lozenges, and nasal sprays [29]. The relapse mechanism enhances the capabilities of the nicotine gene receptors and catalyzers, which act as powerful biological stimulants, thereby increasing the habit of smoking. Consequently, actual nicotine therapies only provide moderate relief. Approximately 80% of patients using one of these medications return to smoking within the first year [29]. Although these pharmacologic treatments may help an individual achieve a relatively long-term abstinence from nicotine use when combined with behavioral interventions, they are limited because they are not tailored to an individual's specific genetic makeup [30].

## 4. CYP2A6 Polymorphism's Impact on Nicotine Dependence

Among the currently identified nicotine gene candidates, the* CYP2A6* gene is the most studied and has been found to be principally responsible for nicotine metabolism, uptake, distribution, and clearance [19]. The* CYP2A6* gene is located on the long arm of chromosome 19 [31].* CYP2A6* belongs to the family of genes known as cytochrome P450, which has a mixed-function oxidase system and is involved in the metabolism of xenobiotics in the body.* CYP2A6* variants have properties that induce or inhibit nicotine according to their structural function [32]; thus, they are hypothesized to enhance or alter nicotine dependence treatment [3].

The evaluation of the enzymatic activities of* CYP2A6* in* in vitro* and* in vivo* experiments has shown that* CYP2A6* polymorphisms can alter or enhance the pharmacokinetics of nicotine according to their structure (deleted/decreased or full function), thus confirming their propensity to predispose to or protect individuals from nicotine dependence [19]. Kubota et al. [2] found that the normal nicotine metabolizer* CYP2A6* ^*∗*^ *1A* gene variant, which has fully functional alleles, encodes the enzyme that metabolizes 100% the volume of inhaled nicotine. Consequently, the CYP2A6^*^1A alleles are associated with a higher daily nicotine intake, indicating that they make the carrier highly susceptible to developing smoking behavior [5].

In an* in vivo* study, Benowitz et al. [33] administered nicotine to clinical trial participants and found that those who carried fully functioning alleles of* CYP2A6* ^*∗*^ *1A* secreted a small quantity of excreted, unchanged nicotine and cotinine N-glucuronide. This quantity represented 25%–30% of the excreted urinary metabolites. They also found a high quantity of cotinine-derived metabolites, consisting of 58%–67% of the excreted urinary metabolites. That experiment provides clues that subjects carrying* CYP2A6* ^*∗*^ *1A* normal nicotine metabolizers are highly susceptible to nicotine dependence. On the other hand, the* CYP2A6* ^*∗*^ *2A* variant is a deleted allele of CYP2A6 gene, and it does not produce enzymes [5]. Therefore, it is inactive and cannot metabolize nicotine [34, 35]. As a result, carriers of CYP2A6^*^2A are poor nicotine metabolizers, smoke less, and recover quickly following nicotine dependence treatment.

In* in vitro* studies, participant carriers of the homozygous* CYP2A6* deleted alleles (*CYP2A6* ^*∗*^ *4*) received nicotine [36]. After two hours, they were found to have very low plasma-cotinine levels and high levels of unchanged nicotine. These different enzymatic activities of the* CYP2A6* polymorphisms result from structural change at the nucleotide level in each of the alleles of* CYP2A6*, as depicted in Table 1. For example,* CYP2A6* ^*∗*^ *1A* is a wild-type allele of* CYP2A6*, which did not evolve; on the other hand,* CYP2A6* ^*∗*^ *1H* to* CYP2A6* ^*∗*^ *12* underwent an evolution or mutation expressed by a change in their nucleotides, thereby affecting their level of enzymatic activities.

## 5. *CYP2A6* Polymorphism Enzymatic Activities and Smoking Behavior

In fact,* CYP2A6* polymorphism consists of alleles (i.e., CYP2A6^*^2A and CP2A6^*^4A) that have a complete loss-of-function or have an enzymatic activity less than 50%, alleles (i.e., CYP2A6^*^9A and CYP2A6^*^12A) with a decrease of function having enzymatic activity less than 75%, and alleles (i.e., CYP2A6^*^1A wild type) that are fully functional with 100% enzymatic activity [26, 33]. The correlation of CYP2A6 alleles' enzymatic activities with the rate of their markers of 3HC/COT production has led to a method to determine smokers' phenotype [37]. Thus, different* CYP2A6* allele enzymatic activities have permitted researchers to classify individual smokers into three CYP2A6 phenotype groups: slow nicotine metabolizers (people who smoke less), intermediary nicotine metabolizers (moderate smokers), and normal metabolizers (heavy smokers) [37], showing an extreme interindividual variability in smoking behavior.  Table 2 summarizes the classification of* CYP2A6* polymorphism according to the patients' enzymatic activities in the two groups.

## 6. *CYP2A6* Polymorphism Impact on Nicotine Dependence Treatment

CYP2A6 polymorphism enzymatic activities have been correlated with nicotine dependence, but their activities also influence the nicotine dependence treatment outcome [33]. In clinical trials, individuals who carried* CYP2A6* variants with diminished enzymatic function activity rates and a low 3HC/COT production ratio while taking bupropion/nicotine replacement therapy (NRT) had the highest cessation rates: 32% compared with those with normal* CYP2A6* allele activities and a high 3HC/COT production ratio, who had only a 10% cessation rate at the end of a 6-month treatment [36]. Using data generated by three clinical trials among Caucasian populations, Patterson et al. [38] demonstrated that individuals using transdermal nicotine patch treatment with a low 3HC/COT ratio and slow CYP2A6  variant nicotine-metabolism activity were significantly more likely to quit smoking than those with high 3HC/COT ratios (indicative of normal CYP2A6 nicotine-metabolism activities) at week eight end-of-treatment (46% as opposed to 28%) and 6 month follow-up visits (30% as opposed to 11%). Therefore, it may be beneficial to translate relevant functions of* CYP2A6* polymorphisms into clinical practice, as they variably convert nicotine to cotinine at 78%, which is further transformed into trans-3-hydroxycotinine (3HC) [39].* CYP2A6* gene variant functions may be harnessed to tailor nicotine dependence treatment for individual treatment needs and should not be neglected in the case of nicotine dependence.

## 7. Nicotine Dependence Treatment One-Size-Fits-All Paradigm

Currently, many new advanced technologies are allowing researchers to understand more complex interactions among genes and genetic interactions with environmental agents [40, 41] and to translate their findings into clinical treatment. In addition, the emerging sciences of pharmacogenetic and pharmacogenomic research have revealed gene functions and the mechanism of their potential clinical utility and validity, which are being incorporated into drug making and disease treatment management [42].

Unfortunately, nicotine dependence treatment programs (cognitive behavioral therapy and pharmacologic therapy) have been rooted in a “one-size-fits-all” paradigm and cannot be readily tailored to the unique needs of individual patients based on their unique constellation of behavioral, biological, and clinical characteristics [43]. Munafò et al. [44] proposed that the one possibility to increase and enhance treatment of nicotine dependence is to offer nicotine dependence treatments tailored to an individual's genotype. Since 70%–80% of inhaled nicotine is biologically converted into cotinine and 90% of this reaction occurs under the influence of the enzyme cytochrome P450* 2A6 *(CYP2A6), the cotinine is then completely transformed by CYP2A6  into trans-3-hydroxycotinin [22].

## 8. Context of Individualized Nicotine Dependence Treatment

**Figure 2:**
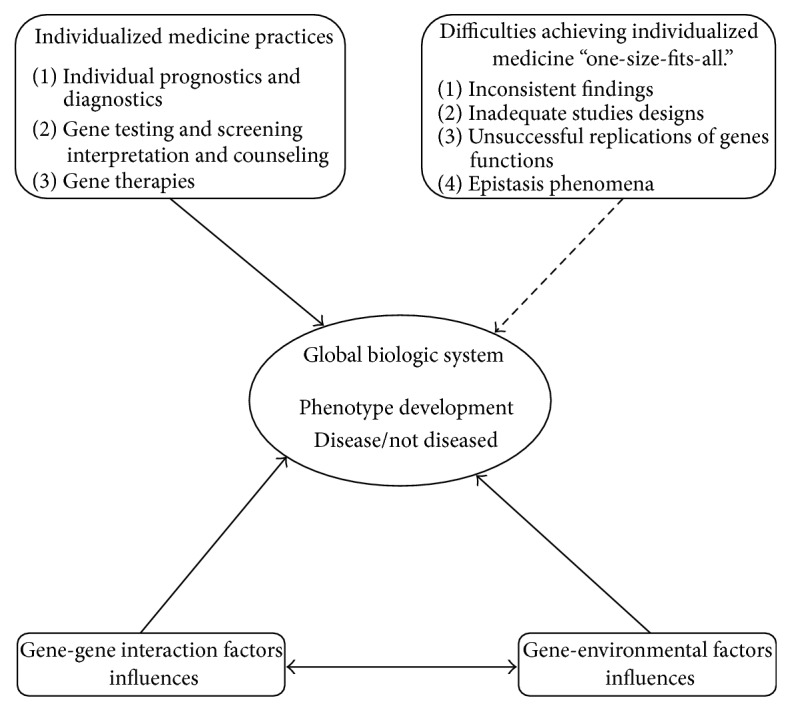
Schema illustrating personalized medicine concept and its difficulties.* Note.* Dashed lines indicate difficulties achieving personalized medicine. The figure is the illustration of classic “one-size-fits-all” pharmacological therapies to individualized treatment which takes to account the individual genotype makeup.

Twin studies have demonstrated that a person's genetic makeup contributing to his or her liability to nicotine, alcohol, and other drug addictions can also shield the individual from the development of addiction [38, 41]. Therefore, treating nicotine patients on an individual basis according to their genotype may help increase a successful nicotine dependence treatment outcome [41]. For example* CYP2A6* polymorphism provides information that governs the basics if biological and physiological activities in nicotine dependence that can be correlated to individual genetic makeups [38]. In addition, the human genome shows that the DNA sequences of any two individuals are 99.9% identical and the 0.1% difference in DNA constitutes the origin of profound genetic material diversity. Therefore, it is very important that nicotine addiction treatment be individualized according to the patient's genetic makeup [38] as summarized in Figure 2 illustrating personalized medicine concept.

A large interindividual variability of nicotine metabolism underlines that the treatment of nicotine dependence should consider information regarding candidate nicotine genes. In a quantitative study, Nakajima et al. [35] compared different alleles of* CYP2A6* genes' nicotine-metabolism activities and found large interindividual variability in the cotinine/nicotine ratios in races with the following means: Caucasian 7.1 ± 4.7; Black 7.2 ± 5.0; Chinese, 8.7 ± 11.9; and Japanese, 3.8 ± 3.1. These different nicotine metabolism activities of CYP2A6 alleles in races confirmed the varying prevalence of nicotine dependence across a race spectrum [18, 22, 45].

## 9. Finding the Impact of CYP2A6 Polymorphisms

To date, few emerging smoking-cessation clinical trials have reported that CYP2A6 activity is associated with nicotine-quitting success [30]. Unraveling the role of gene variants and determining how to use them to design effective therapy are difficult [46] but not unachievable [40].

This goal can be realized through studies that may provide accurate assessment through multivariable analyses of* CYP2A6* genes variants in correlation with nicotine dependence and withdrawal syndrome, other genes, and different forms of therapies [42]. A correlational study could also help examine to what extent* CYP2A6 *major variants are correlated with other nicotine dependence risk factors and how they can influence nicotine dependence treatment.

## 10. Summary

CYP2A6 polymorphisms mediate the metabolism of nicotine according to their ability to encode for the enzyme catalyzer of nicotine to cotinine [13]. They serve as a biological gateway for nicotine to reach the brain by the bloodstream [13]. The fully functional allele CYP2A6^*^1A metabolizes enough nicotine each time a tobacco user inhales the smoke of cigarette to lead to nicotine dependence. In contrast, slow nicotine metabolizers catalyze less nicotine, which prevents addiction [22]. In nicotine dependence treatment, carriers of slow nicotine metabolizer genes are 1.7 times more likely to quit smoking or maintain abstinence than those who have a normal nicotine metabolizer underlining their potential health benefit; these metabolizers can therefore be used in nicotine dependence treatment [30].

The role of* CYP2A6* polymorphism capability to catalyze nicotine provides clues that nicotine treatment should be individualized. Twin, adoption, clinical trial, and case-control studies suggested that there is strong evidence of the influence of genetic variants in nicotine-dependence etiology and treatment, where genetic variants' contributions were evaluated at more than 50% [1, 32], conveying the possibility that* CYP2A6* polymorphism information analysis may strengthen nicotine dependence treatment.

## Supplementary Material

This document presents the definition of main key words and concepts used in this article to assist readers as supplementary definition glossary. The author hopes that these definitions may help for a better comprehension of the article.

## Figures and Tables

**Table 1:** Example of *CYP2A6* gene variants structures and related enzymatic activities in nicotine metabolism.

*CYP2A6* gene variants	Nucleotide change	Protein alteration	Enzymatic activities
*CYP2A* ^*^ *1A* (wild type)	None	None	Normal
*CYP2A6* ^*^ *1H *	-745A>G	Disruption of CCAAT box	Decrease
*CYP2A6* ^*^ *4A *	CYP2A6 deleted	No produced protein	None
*CYP2A6* ^*^ *9A *	-1013A>G, -48T>G; 51G>A	Change in TATA box	Decrease
*CYP2A6* ^*^ *12A *	Change Exon 3–8 of *CYP2A6* origin	10 amino acid substitution	Decrease

Note: change in the nucleotide of these gene variants alters or inhibits their enzymatic activities decreasing their nicotine metabolism activities in comparison with CYP2A6^*^1A (wild type) gene variants [22].

**Table 2:** Estimates of *CYP2A6* gene variants nicotine metabolizer activities.

Metabolizer groups	Estimates enzymatic activities	Genotype descriptions
Normal metabolizer (NM)	100% nicotine metabolism	No loss of alleles function detected *CYP2A6* ^*^ *1A *
Slow metabolizers (SM)	(1) Less than 50% of nicotine metabolism activities	Less than 1/0 allele loss-of-function. Examples: *CYP2A* ^*^ *2* & ^*^ *4 *
	(2) Nearly 75% of nicotine metabolism activities	1 allele loss-of-function Examples: *CYP2A* ^*^ *9 *& ^*^ *12 *

*Note*. In general, gene variant decreases enzymatic activities due to their loss-of-functions [3, 22].

## References

[1] Kortmann G. L., Dobler C. J., Bizarro L., Bau C. H. D. (2010). Pharmacogenetics of smoking cessation therapy. *The American Journal of Medical Genetics, Part B: Neuropsychiatric Genetics*.

[2] Kubota T., Nakajima-Taniguchi C., Fukuda T. (2006). CYP2A6 polymorphisms are associated with nicotine dependence and influence withdrawal symptoms in smoking cessation. *Pharmacogenomics Journal*.

[3] Ho M. K. (2011). *Impact of CYP2A6 genetic variant on nicotine and smoking behavior in light smoking population of Black African descendent [Ph.D. thesis]*.

[4] Mroziewicz M., Tyndale R. F. (2010). Pharmacogenetics: a tool for identifying genetic factors in drug dependence and response to treatment. *Addiction science & clinical practice*.

[5] World Health Organization (2002). *Smoking Statistics—Fact Sheet*.

[6] Center for Disease Control and Prevention (2011). Quitting smoking among adults—United States 2001–2010. *Morbidity and Mortality Weekly Report*.

[7] Uhl G. R., Liu Q.-R., Drgon T. (2008). Molecular genetics of successful smoking cessation: convergent genome-wide association study results. *Archives of General Psychiatry*.

[8] Bassett T. J. (2008). *An Introduction to Behavior Genetics*.

[9] Hall C. S., Stevens S. S. (1951). The genetics of behavior. *Handbook of Experimental Psychology*.

[10] Broadhurst P. L. (1969). Psychogenetics of emotionality in the rat. *Annals of the New York Academy of Sciences*.

[11] Grigorenko E. L., Ravich-Shcherbo I., Grigorenko E. L. (1997). Russian psychogenetics. *Psychology of Russia: Past, Present, Future*.

[12] Fuller J. L., Thompson W. R. (1960). *Behavior Genetics*.

[13] Roses J. R., Broms U., Korhonen T., Dick M. D., Kaprio J. (2009). Genetics of smoking behavior. *Handbook of Behavior Genetics*.

[14] Ring H. Z., Valdes A. M., Nishita D. M. (2007). Gene-gene interactions between CYP2B6 and CYP2A6 in nicotine metabolism. *Pharmacogenetics and Genomics*.

[15] Obach R. S. (2004). Potent inhibition of human liver aldehyde oxidase by raloxifene. *Drug Metabolism and Disposition*.

[16] Al Koudsi N., Hoffmann E. B., Assadzadeh A., Tyndale R. F. (2010). Hepatic CYP2A6 levels and nicotine metabolism: impact of genetic, physiological, environmental, and epigenetic factors. *European Journal of Clinical Pharmacology*.

[17] Tyndale R., Mroziewicz M. The impact of CYP2A6 genotype on smoking cessation in an extended nicotine patch therapy clinical trial. https://tspace.library.utoronto.ca/handle/1807/18929.

[18] Ho M. K., Goldman D., Heinz A. ( 2010). Breaking barriers in the genomics and pharmacogenetics of Drug addiction. *Clinical Pharmacology & Therapeutics*.

[19] Ho M. K., Tyndale R. F. (2007). Overview of the pharmacogenomics of cigarette smoking. *The Pharmacogenomics Journal*.

[20] Lee A. M., Jepson C., Hoffmann E. (2007). *CYP2B6* genotype alters abstinence rates in a bupropion smoking cessation trial. *Biological Psychiatry*.

[21] Remington P. L., Brownson R. C., Wegner M. V. (2010). *Chronic Disease Epidemiology and Control*.

[22] Hukkanen J., Jacob P., Benowitz N. L. (2005). Metabolism and disposition kinetics of nicotine. *Pharmacological Reviews*.

[23] Hughes J. R., Stead L. F., Lancaster T. (2007). Antidepressants for smoking cessation. *Cochrane Database of Systematic Reviews*.

[24] Huang W. L., Li M. D. (2009). Differential allelic expression of dopamine D1 receptor gene (DRD1) is modulated buy microRNA miR-504. *Biological Psychiatry*.

[25] Berrettini W., Yuan X., Tozzi F. (2008). *α*-5/*α*-3 nicotinic receptor subunit alleles increase risk for heavy smoking. *Molecular Psychiatry*.

[26] Johnstone E., Benowitz N., Cargill A. (2006). Determinants of the rate of nicotine metabolism and effects on smoking behavior. *Clinical Pharmacology & Therapeutics*.

[27] MacDougall J. M., Fandrick K., Zhang X., Serafin S. V., Cashman J. R. (2003). Inhibition of human liver microsomal (S)-nicotine oxidation by (−)-menthol and analogues. *Chemical Research in Toxicology*.

[28] Slemmer J. E., Martin B. R., Damaj M. I. (2000). Bupropion is a nicotinic antagonist. *Journal of Pharmacology and Experimental Therapeutics*.

[29] Foulds J. (2006). The neurobiological basis for partial agonist treatment of nicotine dependence: varenicline. *Interactional Journal of Clinical Practice*.

[30] Lerman C., Jepson C., Wileyto E. P. (2010). Genetic variation in nicotine metabolism predicts the efficacy of extended-duration transdermal nicotine therapy. *Clinical Pharmacology & Therapeutics*.

[31] Ingelman-Sundberg M. (2005). Genetic polymorphisms of cytochrome P450 2D6 (CYP2D6): clinical consequences, evolutionary aspects and functional diversity. *Pharmacogenomics Journal*.

[32] Sim C. S. The human cytochrome P450 (CYP) allele nomenclature database. http://www.cypalleles.ki.se.

[33] Benowitz N. L., Swan G. E., Jacob P., Lessov-Schlaggar C. N., Tyndale R. F. (2006). CYP2A6 genotype and the metabolism and disposition kinetics of nicotine. *Clinical Pharmacology and Therapeutics*.

[34] Xu C., Goodz S., Sellers E. M., Tyndale R. F. (2002). CYP2A6 genetic variation and potential consequences. *Advanced Drug Delivery Reviews*.

[35] Nakajima M., Fukami T., Yamanaka H. (2006). Comprehensive evaluation of variability in nicotine metabolism and CYP2A6 polymorphic alleles in four ethnic populations. *Clinical Pharmacology & Therapeutics*.

[36] Yamanaka H., Nakajima M., Nishimura K. (2004). Metabolic profile of nicotine in subjects whose CYP2A6 gene is deleted. *European Journal of Pharmaceutical Sciences*.

[37] Sellers E. M., Tyndale R. F., Fernandes L. C. (2003). Decreasing smoking behaviour and risk through CYP2A6 inhibition. *Drug Discovery Today*.

[38] Patterson F., Schnoll R. A., Wileyto E. P. (2008). Toward personalized therapy for smoking cessation: a randomized placebo-controlled trial of bupropion. *Clinical Pharmacology and Therapeutics*.

[39] National Center of Bioinformatics CYP2B6 cytochrome P450, family 2, subfamily B, polypeptide 6 [ *Homo sapiens* (human) ]. http://www.ncbi.nlm.nih.gov/gene?Db=gene&Cmd=DetailsSearch&Term=1555.

[40] Cohen N. (2008). *Pharmacogenomics and Personalized Medicine*.

[41] Julio L., Ma-Li W. (2002). *Pharmacogenomics. The Searched for Individualized Therapies*.

[42] Khoury J. M., Brosian R. S., Gwinn M. (2010). *Human Genome Epidemiology*.

[43] Nowack R. (2008). Review Article: cytochrome P450 enzyme, and transport protein mediated herb-drug interactions in renal transplant patients: grapefruit juice, St John's Wort—and beyond!. *Nephrology*.

[44] Munafò M. R., Clark T. G., Johnstone E. C., Murphy M. F. G., Walton R. T. (2004). The genetic basis for smoking behavior: a systematic review and meta-analysis. *Nicotine & Tobacco Research*.

[45] Jorde L. B., Wooding S. P. (2004). Genetic variation, classification and ‘race’. *Nature Genetics*.

[46] Carter B. L., Long T. Y., Cinciripini P. M. (2004). A meta-analytic review of the CYP2A6 genotype and smoking behavior. *Nicotine and Tobacco Research*.

