# Supplementary material for: CYP2A6 Polymorphisms May Strengthen Individualized Treatment for Nicotine Dependence
Source: Scientifica (Cairo). 2015 Apr 28;2015:491514. doi: 10.1155/2015/491514 (PMC4427762; doi:10.1155/2015/491514)
Supplement: Supplementary file 1 — This document presents the definition of main key words and concepts used in this article to assist readers as supplementary definition glossary. The author hopes that these definitions may help for a better comprehension of the article. [file 491514.f1.pdf]

Operational Definitions

Allele: One of two or more forms of a genetic locus (Feero, Guttmacher, & Collins, 2010).

Pharmacokinetics: Refers to genetic differences in metabolic pathways that can affect individual responses to drugs, both in terms of therapeutic effect as well as adverse effects (Cohen, 2008).

Pharmacogenomics: The branch of pharmacology that studies the influence of genetic variation on drug response in patients by correlating gene expression or single-nucleotide polymorphisms with a drug's efficacy or toxicity (Wang, 2010).

A single-nucleotide polymorphism (SNP): “A DNA sequence variation due to a single nucleotide—A, T, C or G—change in the genome of paired chromosomes in an individual.” (Varela & Amos, 2010)

Behavioral genetic theory (BGT): “A theory that proposes that genes play a role in human behavior; its primary goal is to establish correlational relationships between genes and behavioral development or disorder etiology.” (Basset, 2008)

Bupropion (Zyban®): A medication used to treat major depressive disorder. The Zyban brand of bupropion is used to help people stop smoking by reducing cravings and other withdrawal effects (Hughes, Stead, & Lancaster, 2007).

Clinical studies: Types of studies examining the distribution of diseases, the factors that affect health, and how people make health-related decisions, or studies seeking to identify the most effective and most efficient interventions, treatments, and services (Khoury et al., 2010).

Clinical validity: Refers to the extent or degree to which a genetic test accurately predicts the risk of a disease outcome (i.e., calibration), as well as its ability to separate patients with different outcomes into separate risk classes (discrimination; Khoury et al., 2010).

Cotinine (COT): An alkaloid and metabolite of nicotine found in tobacco. Cotinine remains longer in body fluid than nicotine. Consequently, cotinine is used as a biomarker to verify or measure exposure to tobacco smoke. Cotinine presence in body fluid also serves as proof of recent nicotine use (The Free Dictionary, 2012).

Cytochrome P450 superfamily (officially abbreviated as CYP): Consists of multiple groups of enzymes that catalyze the oxidation of organic substances. The substrates of CYP enzymes are metabolic intermediates such as lipids, steroidal hormones, and xenobiotic substances such as drugs and other toxic chemicals. CYPs are the main enzymes implicated in drug metabolism and bioactivation (Danielson, 2002).

*Carbon monoxide (CO):*A colorless, odorless, highly poisonous gas formed by the incomplete combustion of carbon or a carbonaceous material such as gasoline (The Free Dictionary, 2012).

Cogintive behavioral therapy (CBT): Consists of advising and motivational interventions that are usually combined with pharmacological therapy to treat nicotine dependence (Ahluwalia et al., 2006).

Deoxyribonucleic acid (DNA): Nucleic acid that contains genetic information needed for the development and the functioning of all living organisms and some viruses. DNA is often considered a set of blueprints that carries instructions to construct other cells, such as proteins and RNA molecules (Forensic Fact, 2008).

Enzyme: A protein (or protein-based molecule) that speeds up a chemical reaction in living organisms. It catalyzes specific chemical reactions and converts a specific set of reactants (called *substrates*) into specific products. (Encyclopedia.com, 2012)

Fagerström Test for Nicotine Dependence (FTND): An instrument used to measure nicotine dependence severity. (Piper et al., 2008)

Genetic variation: Loosely, a measure of the genetic differences within populations or species. For example, a population with many different alleles at a locus may be said to have a lot of genetic variation at that locus (Khoury, et al., 2010).

Genotype: The genetic makeup of a cell, an organism, or an individual (i.e., the specific allele makeup of the individual), usually with reference to a specific character under consideration (Khoury et al., 2010).

Nicotine: An alkaloid found in the nightshade family of plants (*Solanaceae*) that constitutes approximately 0.6%–3.0% of the dry weight of tobacco, with biosynthesis taking place in the roots and accumulation occurring in the leaves (Ho & Tyndale, 2007).

Nicotine dependence: An addiction to tobacco products caused by one of its main ingredients, the drug nicotine, which is a psychoactive or mood-altering drug with pleasurable, stimulant and depressive effects. These effects are temporarily pleasing, making people want to use it more and more, which leads people to switch from experimental smoking to regular nicotine use, and, finally, to nicotine dependence (Remington et al., 2010) .

Phenotype: An observable characteristic or trait such as morphology; development, biochemical, or physiological properties; phenology; behavior; and products of behavior (such as a bird's nest) that result from the expression of an organism's genes as well as the influence of environmental factors and the interactions between the two (Khoury et al., 2010).

Polymorphism: The existence of many forms of DNA sequences at a locus within the population (Wang, 2010).

Personalized medicine: A medical model that emphasizes, in general, the customization of healthcare, with all decisions and practices being tailored to individual patients in whatever ways possible (Cohen, 2008).

Slow metabolizer (SM): CYP2A6 gene variant for a slow metabolizer of nicotine (Benowitz et al., 2006).

Normal metabolizer (NM): CYP2A6 gene variant for a normal metabolizer of nicotine (Benowitz et al., 2006).
